# Supplementary material for: Post-market surveillance of six COVID-19 point-of-care tests using pre-Omicron and Omicron SARS-CoV-2 variants
Source: Microbiol Spectr. 2024 May 17;12(7):e00163-24. doi: 10.1128/spectrum.00163-24 (PMC11218491; doi:10.1128/spectrum.00163-24)
Supplement: Table S1 — Contains limit of detection analysis values for each test. [file spectrum.00163-24-s0002.docx]

**SUPPLEMENTAL TABLE 1.** Probit regression calculated 95% Limit of Detection of six COVID-19 point-of-care tests based on 149 SARS-CoV-2 positive clinical samples.

| **Test** | **Predicted 95% LoD (Ct) 95% CI^1^** | | | | |  |
| --- | --- | --- | --- | --- | --- | --- |
|  | **All (n=149)** | | **Pre-omicron (n=54)** | | **Omicron (n=95)** | |
| **Molecular Tests** | | | | | | |
| ID NOW | 34.7 | (33.1, 36.4) | 34.6 | (32.6, 36.6) | 35.8 | (33.4, 38.2) |
| Check IT | 39.3 | (37.2, 41.4) | N/A^2^ | | 38.7 | (35.6, 41.8) |
| **Rapid Antigen Tests** | | | | | | |
| Panbio | 26.3 | (24.9, 27.6) | 25.9 | (23.8, 27.9) | 26.6 | (24.9, 28.4) |
| Rapid Response | 28.4 | (27.0, 29.7) | 28.7 | (26.8, 30.7) | 28.2 | (26.4, 30.1) |
| QuickVue | 26.6 | (25.2, 28.0) | 25.4 | (23.1, 27.6) | 27.7 | (26.1, 29.3) |
| Biosensor | 25.0 | (23.2, 26.8) | 26.1 | (23.4, 28.8) | 24.4 | (21.9, 26.9) |

^1^95% Wald confidence intervals; ^2^N/A since insufficient data, n - number of SARS-CoV-2 positive samples.
